# Endotoxin induced acute kidney injury modulates expression of AQP1, P53 and P21 in rat kidney, heart, lung and small intestine

**DOI:** 10.1371/journal.pone.0288507

**Published:** 2023-07-25

**Authors:** Wuyang Lv, Longge Xue, Lei liang, Dongyang Liu, Cuicui Li, Jia Liao, Yingyu Jin

**Affiliations:** Department of Clinical Laboratory, the First Affiliated Hospital of Harbin Medical University, Harbin, Heilongjiang, China; Gifu University School of Medicine Graduate School of Medicine: Gifu Daigaku Igakubu Daigakuin Igakukei Kenkyuka, JAPAN

## Abstract

This study was designed to explore whether aquaporin 1(AQP1), P53 and P21 can be used as diagnostic biomarkers of lipopolysaccharide (LPS)-induced acute kidney injury (AKI) and potential indicators of sepsis-induced multiple organ injury. Bioinformatics results demonstrated that AQP1, P53, P21 was dramatically elevated 6h after Cecal ligation and puncture (CLP)-AKI in rat renal tissue. The expression of AQP1, P53, P21, NGAL and KIM-1 in kidney were increased significantly at first and then decreased gradually in LPS-induced AKI rats. Histopathological sections showed swelling of tubular epithelial cells and destruction of basic structures as well as infiltration of numerous inflammatory cells in LPS-induced AKI. Moreover, the expressions of AQP1, P53 and P21 in heart were significantly increased in LPS treatment rats, while the AQP1 expressions in lung and small intestine were significantly decreased. The level of NGAL mRNA in heart, lung and small intestine was firstly increased and then decreased during LPS treatment rats, but the expression of KIM-1 mRNA was not affected. Therefore, our results suggest that AQP1, P53 and P21 is remarkably upregulated in LPS-induced AKI, which may be considered as a potential novel diagnostic biomarker of Septic AKI. NGAL may serve as a biomarker of sepsis-induced multiple organ damage during the process of LPS-induced AKI.

## Introduction

Sepsis is a threatening life clinical syndrome characterized by systemic inflammation, protracted host immunosuppression and multiple organ dysfunction caused by a patient’s dysregulated response to infection [1]. One of the most vulnerable organs during sepsis is the kidney, resulting in sepsis-associated acute kidney injury (SA-AKI), with high morbidity and mortality [1]. Accumulating evidences suggest that pathogenesis of septic AKI is multifactorial and complex, involving inflammation, coagulation cascade activation, oxidative stress, microcirculatory disturbance, and renal venous congestion. Although these studies, the specific diagnostic biomarker in SA-AKI is still absence. Therefore, it is urgent to find novel diagnostic biomarkers to help diagnose sepsis AKI as early as possible, distinguish different types of AKI injury, predict the duration of injury, monitor efficacy and evaluate prognosis, as well as prevent the progression to CKD after AKI.

AQP1, a member of the aquaporin family, is a transmembrane protein whose main role is to transport water molecules [2]. Recently, accumulating researches have shown that AQP1 is involved in the pathogenesis and development of a variety of organ or system diseases, such as cardiovascular disease and kidney disease [3]. AQP1 is distributed in the apical and basolateral plasma membrane of the proximal tubule, descending thin limbs of Henle and descending vasa recta, which is involved in urine concentration and maintenance of body water balance [4]. In our previous animal experiments, AQP1 and NGAL in urine of rats with non-oliguria acute renal injury induced by disseminated intravascular coagulation were detected, and found that AQP1 in urine increased earlier than NGAL, and the magnitude of the increase was more obvious than NGAL, suggesting that AQP1 may be used as a diagnostic marker of AKI. Unfortunately, the expression of AQP1 in renal tissue and plasma of rats could not be detected simultaneously.

P53, a tumor suppressor protein, which is closely related to the pathogenesis of cancer [5]. P53 can be induced by a large number of intracellular/extracellular stimuli and stresses, and acts as a transcription factor to regulate the expression of a variety of downstream genes, thus helping the cell/organism to resist stimuli and injury [6]. In 2003, Dagher and colleagues first found that the expression of P53 was significantly increased during renal injury in ischemic AKI, indicating that P53 may be involved in the pathogenesis of renal disease [7]. Recently, accumulating researches have demonstrated that P53 plays a key role in the pathogenesis of different types of AKI [8, 9]. Ying Y [10] et al. have demonstrated that P53 inhibitor or knockout of P53 significantly reduce the inflammation and epithelial cell apoptosis in the kidney of AKI rats induced by ischemia-reperfusion. P21 is a critical cell cycle regulatory protein which promotes cell cycle arrest by inhibiting multiple cyclin dependent kinases, most notably, CDK2 [11]. P21 can behave as a renal “stress” protein, being up-regulated by diverse forms of AKI [12–14]. Moreover, P21 is also a downstream factor of P53, as one of the markers of P53 activation [15]. Interestingly, the researchers demonstrated in a mouse model of pulmonary hypertension that AQP1 expression was increased in pulmonary artery smooth muscle cells, thereby promoting cell proliferation and exacerbating disease progression, while the expression of P53 in pulmonary artery smooth muscle cells was decreased [16, 17]. In contrast, P53 expression was significantly increased when AQP1 was knocked down, suggesting that there may be some association between P53 and AQP1. Similarly, Kandemir FM [18] and colleagues demonstrated that inhibition of P53 significantly attenuated renal injury and increased renal AQP1 expression in vancomycin-induced AKI rats, which further supports the hypothesis of an association between AQP1 and P53 proteins.

In brief, researchers have shown that the expression of P53 and AQP1 is changed in the kidney after AKI, and the inhibition of P53 or up-regulation of AQP1 has a renal protective effect, suggesting that AQP1 and P53 may be involved in the pathological mechanism of AKI. However, there are still few studies on AQP1, P53 and P21 as diagnostic markers of septic AKI. Therefore, the purpose of this study was to explore whether AQP1, P53 and P21 in kidney, serum and urine of rats can be used as diagnostic markers of septic AKI or potential indicators of sepsis-induced multiple organ injury.

## Materials and methods

### Acquisition and preprocessing of gene microarray data

We searched the public microarray database GEO (Gene Expression Omnibus, http://www.ncbi.nlm.nih.gov/geo/) for AKI-related samples. Raw data (Excel file) from GSE109041 were downloaded from the GEO database. GSE109041 derived total kidney mRNAs from rat with Cecal ligation and puncture (CLP)-induced AKI. The robust multiarray averaging method was then used to preprocess the raw data. Probes were annotated with the latest official annotations file. All bioinformatics analyses were processed and analyzed by GraphPad Prism (8.0.1).

### Septic AKI model establishment

Male Wistar rats, weighed between 180–220 g and aged 8–9 weeks, were obtained from the Laboratory Animal Center of Harbin Medical University, Heilong jiang, China. This study protocol was reviewed and approved by the Harbin Medical University Institutional Animal Ethics Committee, approval number 2021089. All animals were housed in individual cages under controlled environment with temperature control (22–24°C). The rats had free access to water and food. LPS-induced AKI model was induced with 10mg/kg LPS via intraperitoneal injection [19]. All rats (n = 42) were randomly assigned into one of the two experimental groups: the control group (n = 21), injected intraperitoneally with 600μl of 0.9% saline, or the LPS group (n21), injected intraperitoneally with 10mg/kg body weight LPS (Escherichia coli serotype 0111: B4, Sigma Aldrich, USA, 10mg/kg LPS dissolved in 600μl 0.9% normal saline) solution. According to the time points after liquid injection (8h, 12h, 24h, 48h and 72 h, 7d, 14d), each group was further divided into seven subgroups (n = 3/group), and the rats were then observed and treated at different study periods.

Rats were anesthetized by intraperitoneal injection of 3% pentobarbital sodium solution (CAS number:57-33-0, Sigma-Aldrich, USA, 0.2ml/100g body weight), then the chest was opened, and blood was collected by cardiac puncture using a 5ml sterile syringe. The serum was separated by centrifugation at room temperature at 3500 rpm for 10min. The levels of SCr and BUN were analyzed using an automatic biochemical analyzer (Vitros V5600, Johnson, USA). A 1.5-fold increase in serum creatinine compared with the control group demonstrates a successful establishment of the LPS induced AKI model. The remaining serum was stored in sterile EP tubes at -80°C for subsequent experiments. Next, the abdominal cavity was opened, and urine was collected by bladder puncture using a 1ml sterile syringe and frozen at -80°C. The two kidneys, heart, lungs, small intestine were removed and washed with sterile saline. The left kidney, heart, lungs, small intestine was frozen at -80°C (for western blot analysis and RT-qPCR assay), and the right kidney was immersed in 4% paraformaldehyde and stored at 4°C for hematoxylin and eosin (HE) staining.

### HE staining of renal tissue and renal injury scoring

The right kidneys were removed from the rats and fixed with 4% paraformaldehyde for 24h, followed by embedding in Optimum Cutting Temperature (OCT) compound (SAKURA, San Francisco, CA, USA) for evaluation of kidney tubular cell damage and inflammatory exudation [20]. Renal sections of 4μm thickness were stained with HE using standard procedures.

### Measurement of AQP1, P53 and P21 in plasma and urine

The concentration of AQP1, P53 and P21 in plasma and urine were detected with rat ELISA kits (Jianglai Biology, Shanghai, China). The procedures were performed according to the manufacturers’ instructions. Absorbance was detected at 450 nm by an automated ELISA reader (MULTISKAN FC, Thermo, MA, USA). Concentrations in the samples were calculated using a standard curve and results are expressed in pg/ml.

### Western blot analysis

The kidneys, heart, lungs and small intestine were removed from -80°C degrees and slowly thawed on ice. 30mg of kidneys, heart, lungs and small intestine tissue were weighed and added with pre-cooled 200μl RIPA buffer (Solarboi life Sciences, Beijing, China) and 2μl PMSF (100mM) (Solarboi life Sciences, Beijing, China), which was ground on ice and lysed. Proteins in the supernatants were extracted and quantified by BCA assay. Add 5× loading buffer (Solarboi life Sciences, Beijing, China) to the extracted protein supernatant and boil for 10min and proceed to the next step. After electrophoresis on 12% SDS-PAGE gels, the proteins were blotted to polyvinylidene fluoride (PVDF) membranes (Millipore-Upstate). After blocking in nonfat milk (5%) for 60minutes at room temperature, the membranes were incubated at 4°C for 12-18h with antibodies, including AQP1(ab168387; 1:1000; Abcam, USA), P53(ab26; 1:1000; Abcam, USA), P21(ab109520; 1:1000; Abcam, USA) and β-actin (1:1000; Zhongshan jinqiao Biological; China). After washing three times with Tris-buffered saline with Tween 20 (TBST) at room temperature, each of 10 min, the membranes were incubated with secondary antibodies, including a goat anti-Rabbit IgG (1:10000; Proteintech, Hubei, China) a goat anti-mouse IgG (1:10000; Abcam) at room temperature for 1h. Proteins were visualized using enhanced chemiluminescence-plus reagents (No: WB100D, NCM Biotech). The density of the bands was measured using the Image J software (version 1.45s; National Institutes of Health, Bethesda, MA, USA) and values were normalized to the densitometric values of β-actin.

### RNA extraction and quantitative real-time PCR

The kidneys, heart, lungs and small intestine were removed from -80°C below zero and 50mg of tissue was accurately weighed. Total RNA was extracted from rat kidneys, heart, lungs and small intestine tissues using RNAiso Plus reagent (Code No.9109, Takara) according to the manufacturer’s protocol. Extracted and purified total RNA(1μg) was reverse-transcribed into cDNA using a Script cDNA Synthesis Kit (Code No. RR036A, PrimeScript^™^RT Master Mix, Takara) at 37°C for 15min and at 85°C for 5 sec. The total system of reverse transcription was 10μl. The real-time quantitative PCR reaction volume was 20μl and included cDNA, dNTPs, primers, SYBR Premix Ex Taq (Perfect Real Time; Takara) and nuclease-free water. The following qPCR conditions were used for AQP1, P53, P21, NGAL, KIM-1 and GAPDH: 40 cycles of denaturation at 95°C for 5s, annealing at 60°C for 30s, and extension at 72°C for 15s. Results were calculated using the 2^−ΔΔCt^ method and each experiment was performed in triplicate. Primer sequences used are provided in Table 1.

**Table 1:** Primer sequences of Wistar male rats.

The purpose gene	Primer sequence (5’-3’)
**AQP1**	Forward Primer ACCTGCTGGCCATTGACTAC
	Reverse Primer CCAGGGCACTCCCAATGAAT
**P53**	Forward Primer CGAGATGTTCCGAGAGCTGAATG
	Reverse Primer GTCTTCGGGTAGCTGGAGTG
**P21**	Forward Primer CGGGCAGTCCCTTCTAGTTCC
	Reverse Primer AATGCTTGAGCACACACGAG
**NGAL**	Forward Primer CCGACACTGACTACGACCAG
	Reverse Primer CATTGGTCGGTGGGAACAGA
**KIM-1**	Forward Primer GCCTGGAATAATCACACTGTAAG
	Reverse Primer GCAACGGACATGCCAACATAG
**GAPDH**	Forward Primer AAGGGCTCATGACCACAGTC
	Reverse Primer GGATGCAGGGATGATGTTCT

### Assessment of the degree of renal injury

Two professional pathologists, who were blinded to the experimental protocol and the experimental group of this subject, scored the damage of the renal cortex. Three sections were selected for each group, and 10 random fields of view were selected from each section. The histological assessment of renal damage included the observed loss of the tubule brush border, renal tubule swell, renal tubule hemorrhage, tubular casts, and obvious inflammatory infiltration in the cortex [21, 22]. The degree of renal injury in the cortex was determined using a semiquantitative grade scale, where 0 = no abnormality, 1 = minimal damage (involvement of less than 25% of cortex), 2 = mild damage (25%-50%), 3 = moderate damage (50%-75%), and 4 = severe damage (>75%) [23]. Finally, statistical analysis and graphing were performed, according to the scoring results.

### Statistical analysis

Statistical analyses were carried out using GraphPad Prism 8.0.1 software (La Jolla, CA, USA). The data are expressed as the mean ± standard error of the mean (SEM). The treatment effects were analysed by using one-way analysis of variance (ANOVA) and two-tailed Student’s t tests. Specifically, comparisons among multiple groups were conducted by one-way analysis of variance (ANOVA) and homogeneity test of variance. The two-tailed Student’s t test was used for the comparison of two groups. *p<0.05 was considered to be reflective of statistical significance.

## Results

### The microarray analysis results of septic AKI

**Fig 1:**
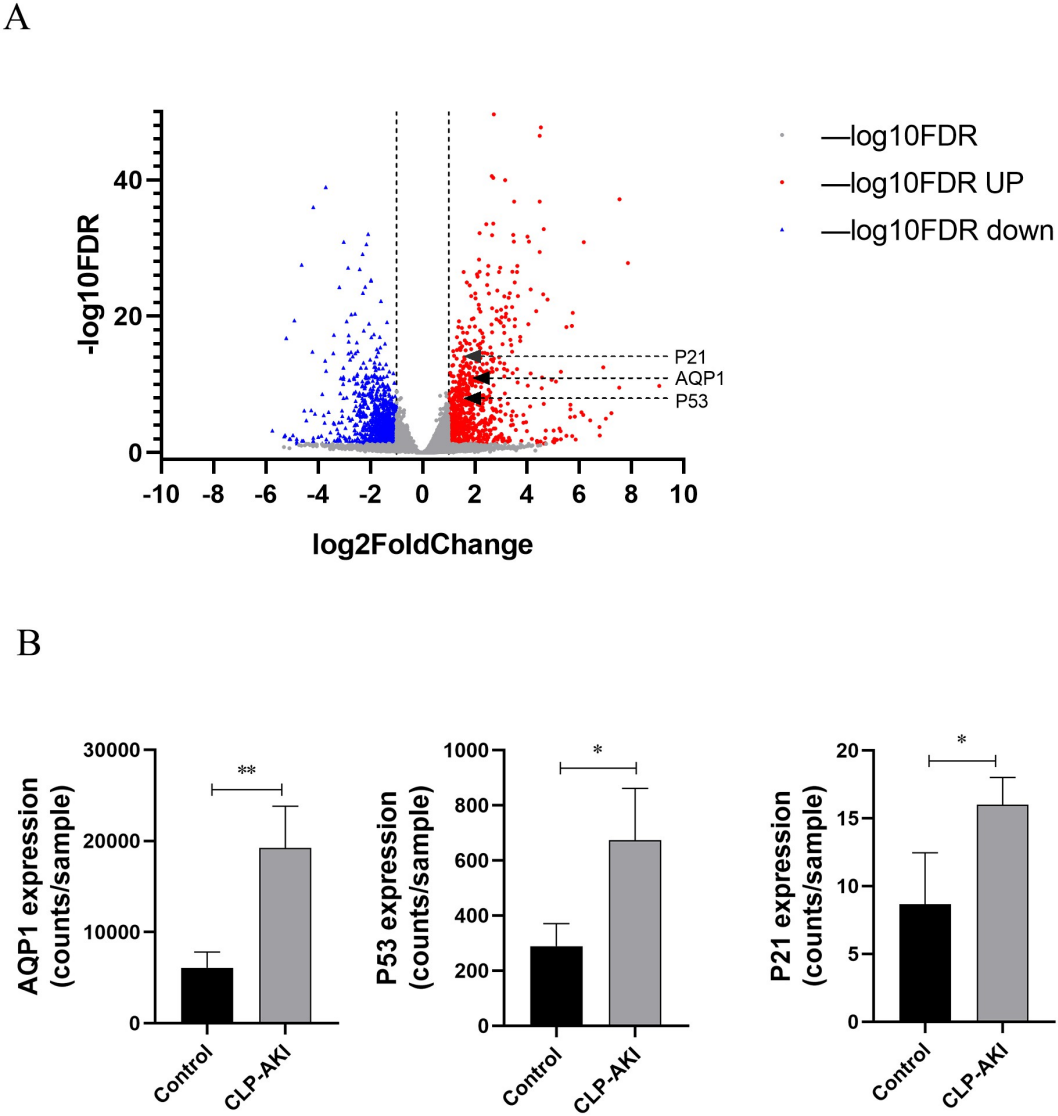
The microarray analysis results of septic AKI. (A) Gene expression profiling of kidney at 6h after CLP-induced AKI compared with the control group. Volcano plot of log2 fold changes versus -log10 FDR showed transcriptional differences between the CLP-induced AKI group and the control group. Vertical dashed lines denote the cutoff of 2-fold change, and the horizontal dashed line represents the 0.05 FDR cutoff. AQP, P53and P21 was increased after 6 h renal CLP-induced AKI in Rat (fold change>2, FDR<0.05). (B) The expression of AQP, P53 and P21 in kidney at 6h after CLP induced AKI. Data are expressed as the mean ± SE. *p < 0.05, **p < 0.01.

There were 1905 different genes at 6h after Cecal ligation and puncture (CLP)-induced AKI in GSE109041 (Fig 1A). And most importantly, AQP1, P53 and P21 were significantly up-regulated in the gene expression profiling of rat kidney at 6h after CLP-induced AKI compared with the control group (fold change>2, FDR<0.05), which suggested that AQP1, P53 and P21 might be connected with AKI and might be a promising biomarker of the Septic AKI (Fig 1A and 1B).

### Assessment of renal function at different time points of endotoxemia AKI

**Fig 2:**
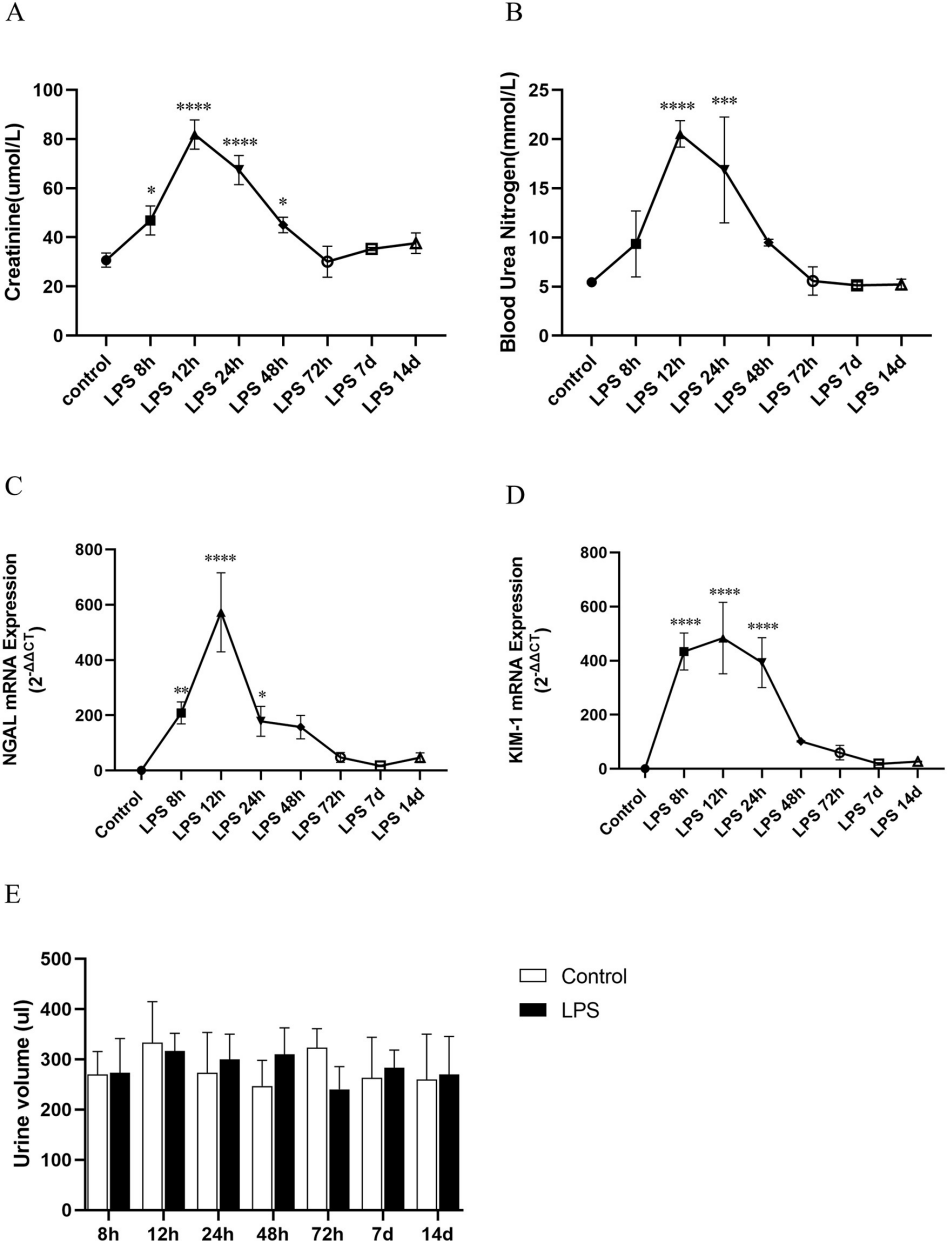
Evaluation of renal function in LPS induced AKI. (A, B) SCr and BUN levels at different times in septic AKI. (C, D) NGAL mRNA and KIM-1 mRNA expression at different time points in septic AKI. (E) Urine volume of rats at different time points. *p < 0.05, **p < 0.01, ***p < 0.001, ****p < 0.0001.

Compared with the control group, SCr and BUN increased significantly at 8h after LPS intraperitoneal injection, and reached the peak at 12h (Fig 2A and 2B), and the SCr and BUN values in LPS group were 3 times the baseline level of the control group, indicating that LPS successfully induced AKI in rats. However, the levels of SCr and BUN decreased gradually at 24h, 48h, and 72h after LPS stimulation, and returned to baseline level at 7d (Fig 2A and 2B). Interestingly, SCr increased slightly at 14d after LPS treatment, which corresponding to be the stage of renal post-AKI conversion to CKD. Moreover, NGAL and KIM-1 are considered as early indicators of renal injury and stress [24]. RT-qPCR detected that NGAL mRNA and KIM-1 mRNA expressions in the kidney of rats were consistent with the change trend of SCr and BUN values in the LPS group (Fig 2C and 2D), which further supported our experimental results. Interestingly, there was no statistically significant change in urine volume between control group and LPS group at different time points (Fig 2E).

### Renal histology and structure of septic AKI

**Fig 3:**
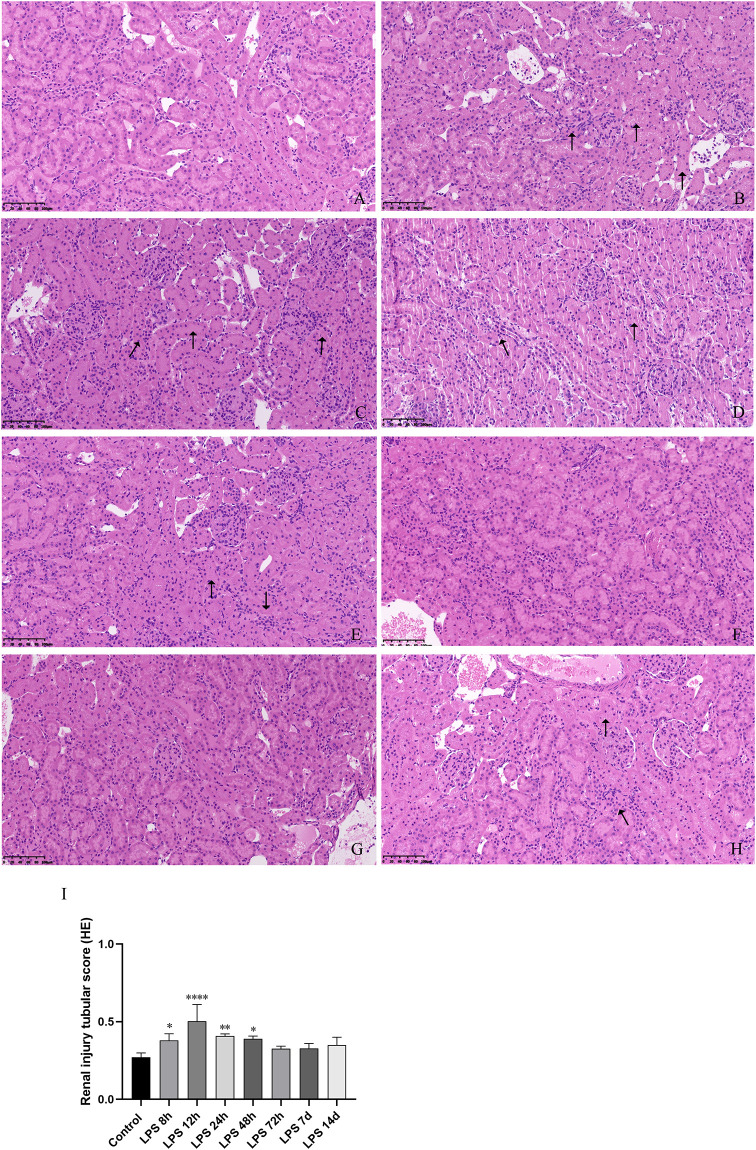
The morphology and structure of renal tissues of control group and LPS group were observed by HE staining (20X). (A)The control group. (B, C, D, E, F, G, H) Rats were treated with LPS for 8h,12h,24h,48h,72h,7d,14d. (I) The tubular damage score was evaluated based on pathological observations from HE staining. *p < 0.05, **p < 0.01, ***p < 0.001, **** *p* <0.0001.

To further observe the extent of renal tissue injury in rats, morphological and structural changes of renal tissue were assessed by HE staining. The kidney histopathological images indicated that the histological structure was disordered, renal tubular epithelial cell edema, tubular lumen narrowing and the renal tissue was infiltrated with a large number of inflammatory cells in the LPS group, while the kidney tissue was not histologically altered in the control group (Fig 3A–3H). These pathological changes gradually aggravated at (8h,12h, 24h and 48h) after LPS intraperitoneal injection compared the control group, and the renal tubular injury score was correspondingly elevated (Fig 3I). This was consistent with the results of SCr and BUN.

### The expression of AQP1, P53 and P21 in serum, urine and kidney in septic AKI

**Fig 4:**
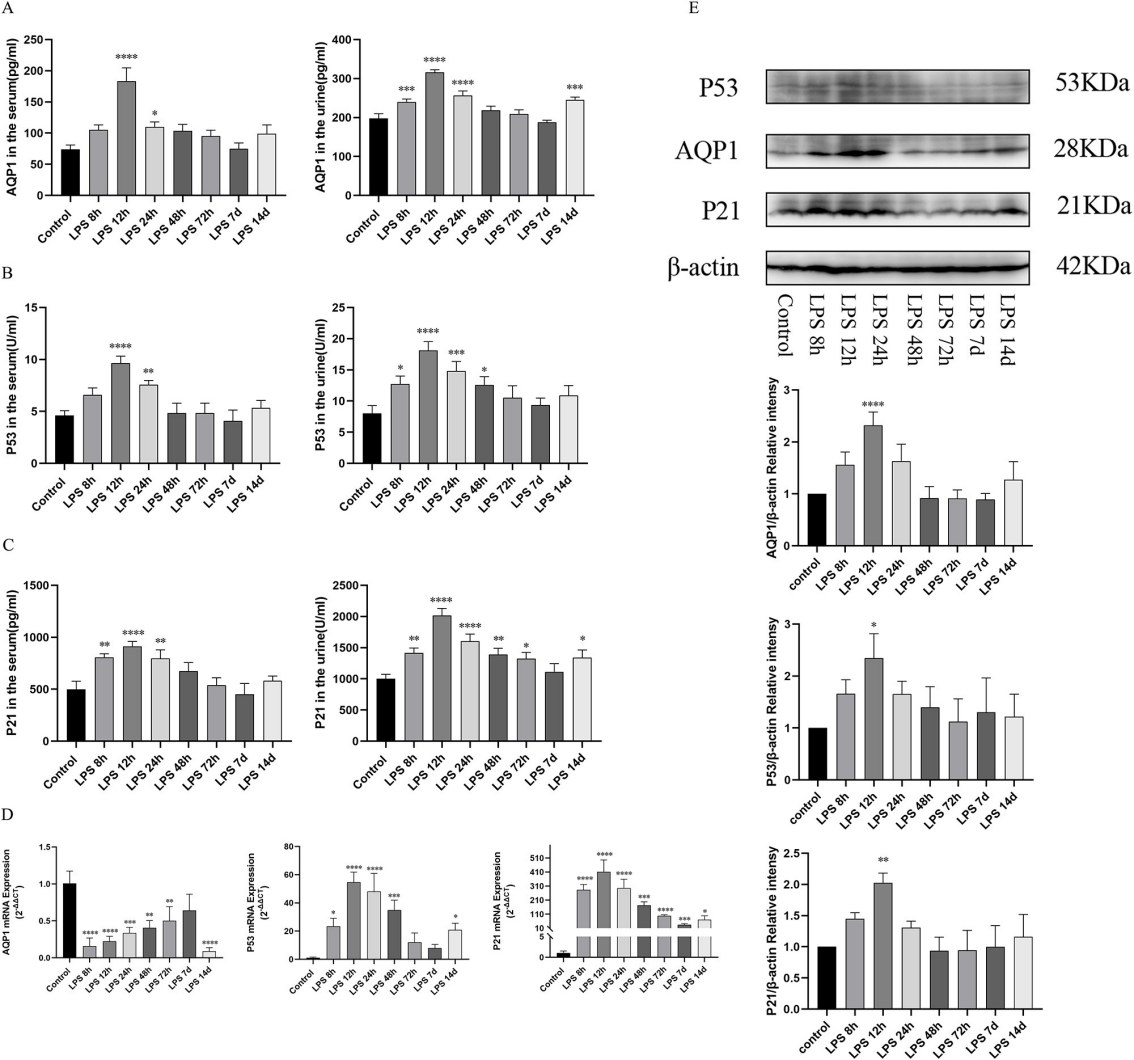
Expression of AQP1, P53 and P21 in serum, urine and renal tissues of rats during the course of LPS-induced AKI. (A, B, C) AQP1, P53 and P21 levels in serum and urine. (D) Expression of AQP1mRNA, P53mRNA and P21mRNA in renal tissue. (E) Protein expression of AQP1, P53 and P21 in renal tissue. *p < 0.05, **p < 0.01, ***p < 0.001, ****p < 0.0001.

ELISA kits were used to detect the concentrations of AQP1, P53 and P21 in plasma and urine of LPS group. Results showed that AQP1 protein expressions in serum and urine were significantly increased at 12h after LPS treatment and gradually decreased at (24h, 48h, 72h, 7d) after LPS treatment (Fig 4A). Meanwhile, we detected the expression changes of P53 and P21 in rat serum and urine. Surprisingly, the expressions trend of P53 and P21 in serum and urine during LPS-induced AKI was consistent with that of AQP1 expression (Fig 4B and 4C). In addition, western blotting showed that the protein expressions of AQP1, P53 and P21 in rat renal tissue increased significantly and reached a peak at 12h after LPS injection and then decreased at other time periods (24h, 48h, 72h, 7d) during the course of endotoxin-induced AKI (Fig 4E), which consistent with the levels of AQP1 in serum. Interestingly, renal AQP1 mRNA levels were dramatically decreased in LPS-induced AKI rats following the increased expression of P53 mRNA and P21 mRNA (Fig 4D). This result suggests that the increased expression of AQP1 protein in renal tissue during septic AKI may be caused by stress or other factors, independent of genetic changes, while the increased expression of P53 and P21 proteins is dependent on genetic changes.

### Expression of AQP1, P53, P21 protein and gene as well as NGAL and KIM-1 mRNA in heart in LPS-induced AKI

**Fig 5:**
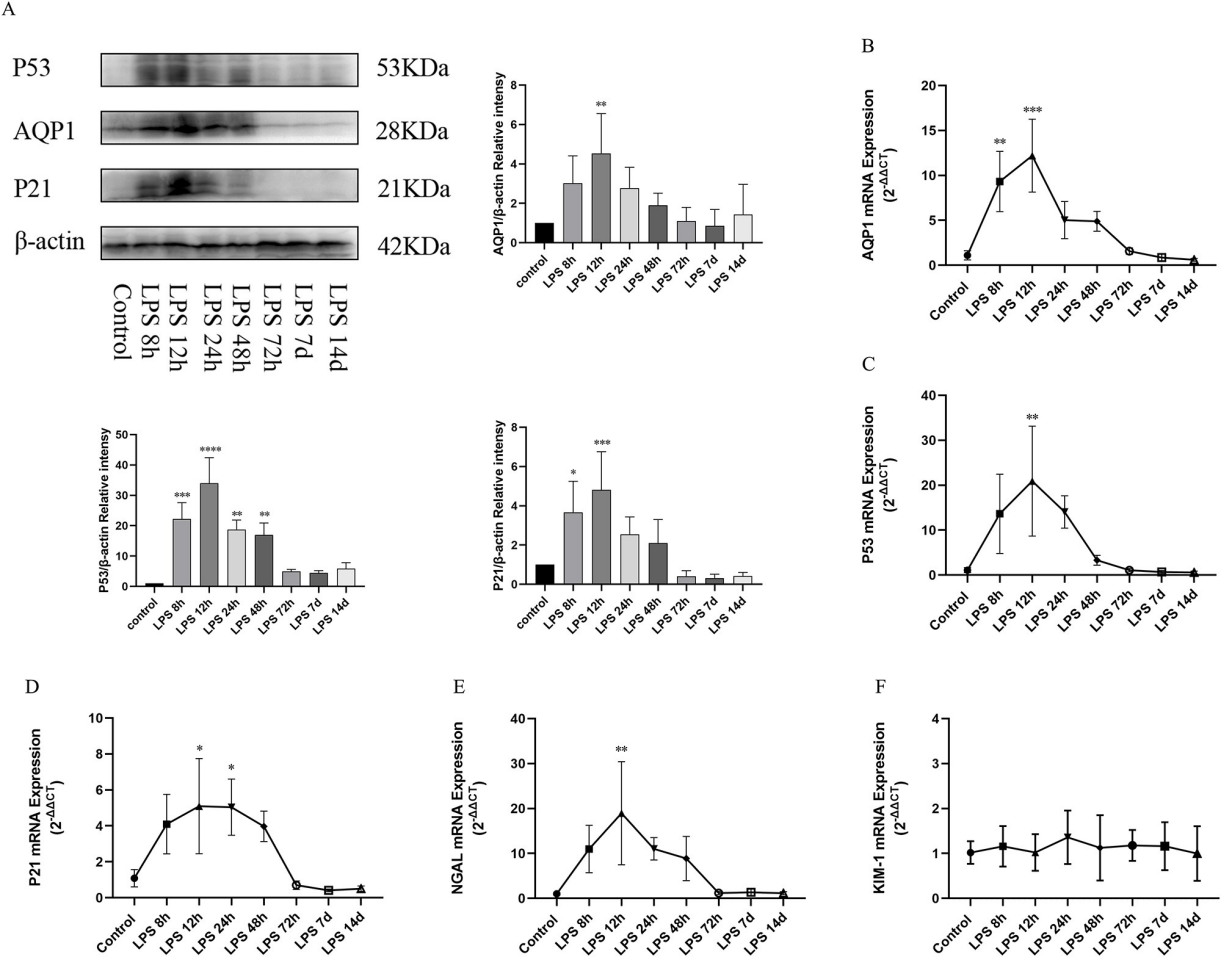
Expression of AQP1, P53, P21 protein and gene as well as NGAL and KIM-1 mRNA in heart during the process of LPS-induced AKI. (A) Expression of AQP1, P53 and P21 proteins in the heart of rats at different time courses after LPS treatment. (B, C, D) Expression of AQP1 mRNA, P53 mRNA, P21 mRNA expression in rat heart. (E, F) Expression of NGAL mRNA and KIM-1 mRNA in rat heart. *p < 0.05, **p < 0.01, ***p < 0.001, ****p < 0.0001.

Multiple organ dysfunction is one of the typical features of endotoxemia. Therefore, we also continued to explore the expression of AQP1, P53 and P21 in other organs besides the kidney in the experimental rat model of sepsis. Western blotting showed that the expression levels of AQP, P53 and P21 in heart of rats with sepsis were significantly increased at (8h,12h,24h,48h) after LPS treatment and dramatically decreased at 72h,7d and14d (Fig 5A). In addition, the mRNA levels of AQP1, P53, and P21 in the heart were consistent with the protein expression changes at different times of LPS treatment (Fig 5B–5D). Next, we also detected the expression of NGAL mRNA and KIM-1 mRNA in the heart. Surprisingly, NGAL mRNA was significantly altered but KIM-1mRNA was unchanged in the heart at different times in the LPS group compared with the Control group (Fig 5E and 5F). Compared with the control group, NGAL mRNA levels in the heart increased significantly at 8h after LPS treatment and peaked at 12h and gradually decreased at 24h, 48h, 72h, 7d, and 14d (Fig 5E). This result was consistent with that of NGAL in the kidneys of LPS group.

### Expression of AQP1, P53, P21 protein and gene as well as NGAL and KIM-1 mRNA in lung during the process of LPS-induced AKI

**Fig 6:**
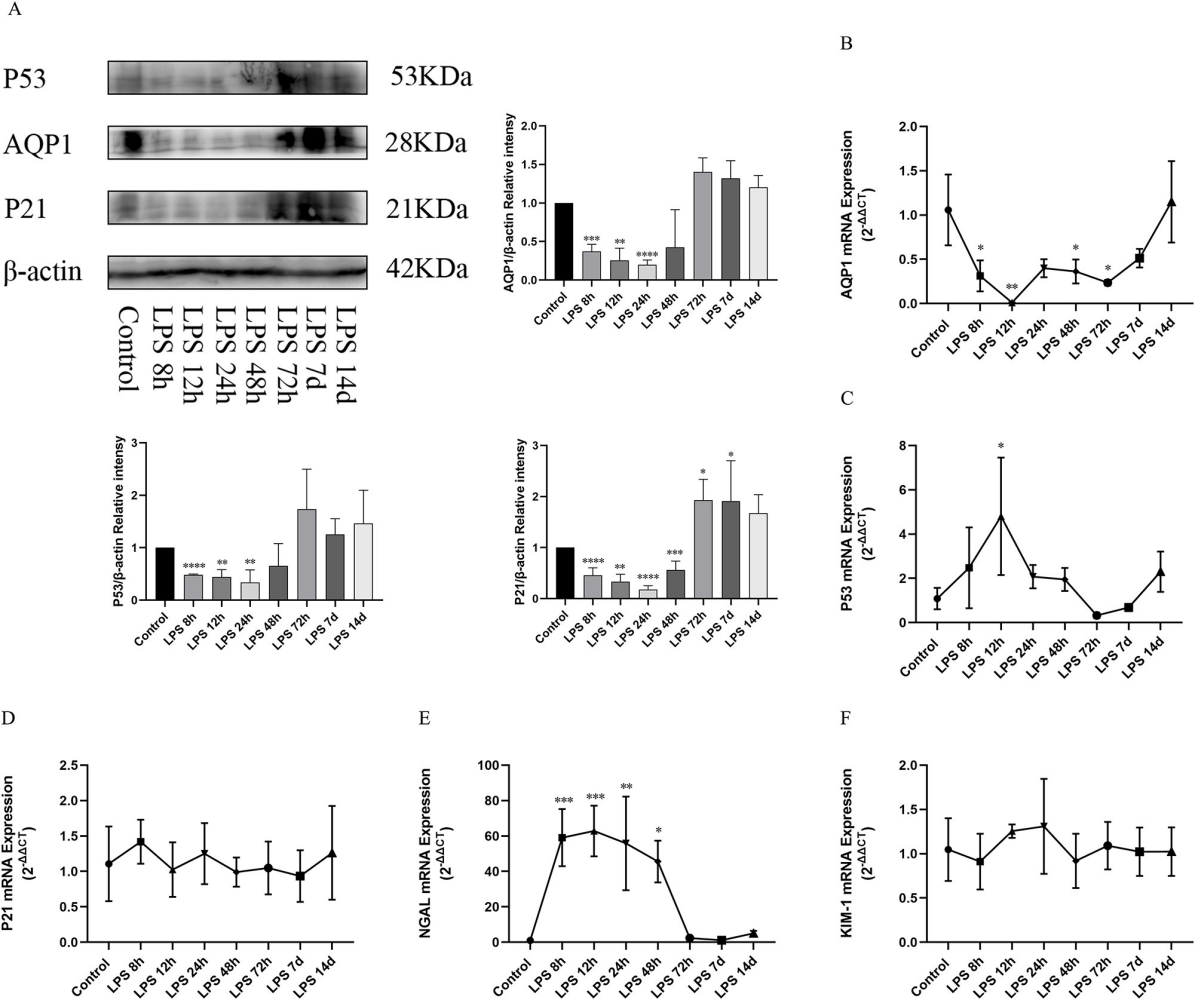
Expression of AQP1, P53, P21 protein and gene as well as NGAL and KIM-1 mRNA in lung during the process of LPS-induced AKI. (A) Expression of AQP1, P53 and P21 proteins in the Lung of rats at different time courses after LPS treatment. (B, C, D) Expression of AQP1 mRNA, P53 mRNA and P21 mRNA in rat lung. (E, F) Expression of NGAL mRNA and KIM-1 mRNA in rat lung. *p < 0.05, **p < 0.01, ***p < 0.001, ****p < 0.0001.

Western blotting showed that AQP1, P53 and P21 protein levels in lung of rats treated with LPS at (8h,12h,24h,48h) were significantly decreased and recovered at (72h, 7d, 14d) compared with the control group (Fig 6A). Moreover, RT-qPCR showed that lung AQP1mRNA expression was decreased at (8h,12h,24h,48h) after LPS treatment and recovered at 14d compared with the control group (Fig 6B). Interestingly, the expression of P53 mRNA in lung increased first and then decreased gradually and the expression of P21 mRNA did not change (Fig 6C and 6D) at different times in LPS stimulation. In addition, NGAL mRNA was significantly altered in the lungs at different times in the LPS group compared with the control group. As shown in Fig 6E, NGAL mRNA in lung significantly increased at 8h after LPS treatment and peaked at 12h, and gradually decreased at 24h, 48h, 72h, 7d and 14d. However, KIM-1mRNA did not change (Fig 6F).

### Expression of AQP1, P53, P21 protein and gene as well as NGAL and KIM-1 mRNA in small intestine during the process of LPS-induced AKI

**Fig 7:**
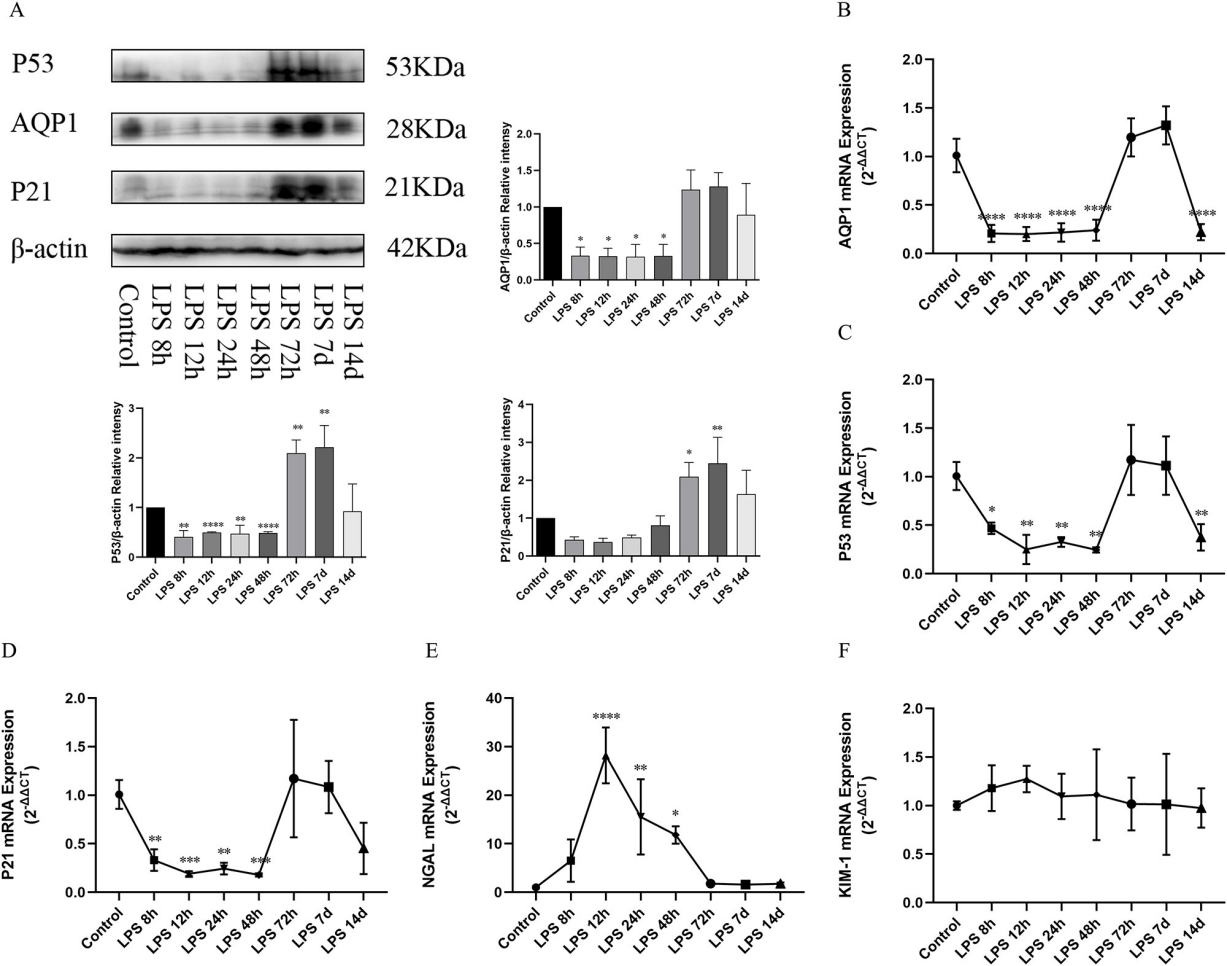
Expression of AQP1, P53, P21 protein and gene as well as NGAL and KIM-1 mRNA in small intestine during the process of LPS-induced AKI. (A) Expression of AQP1, P53 and P21 proteins in the small intestine of rats at different time points after LPS treatment. (B, C, D) Expression of AQP1 mRNA, P53 mRNA and P21 mRNA in rat small intestine. (E, F) NGAL mRNA and KIM-1 mRNA expression in rat small intestine. *p < 0.05, **p < 0.01, ***p < 0.001, ****p < 0.0001.

RT-qPCR and Western blotting analysis was used to showed that the expression of AQP1, P53 and P21 gene and protein in small intestine of rats significantly decreased at (8h,12h,24h,48h) after LPS intraperitoneal injection and recovered at 72h and 7d compared with the control group, but decreased again at 14d after LPS treatment (Fig 7A–7D). Moreover, consistent with the heart and lung results, NGAL mRNA expression in the small intestine was significantly increased at (8h,12h,24h,48h) after LPS treatment and recovered at (72h,7d, and 14d) compared with the control group (Fig 7E). Meanwhile, the expression of KIM-1 mRNA in the small intestine did not change during the course of LPS treatment (Fig 7F).

### Correlation analysis of SCr and BUN with AQP1, P53 and P21 proteins in serum and urine

**Fig 8:**
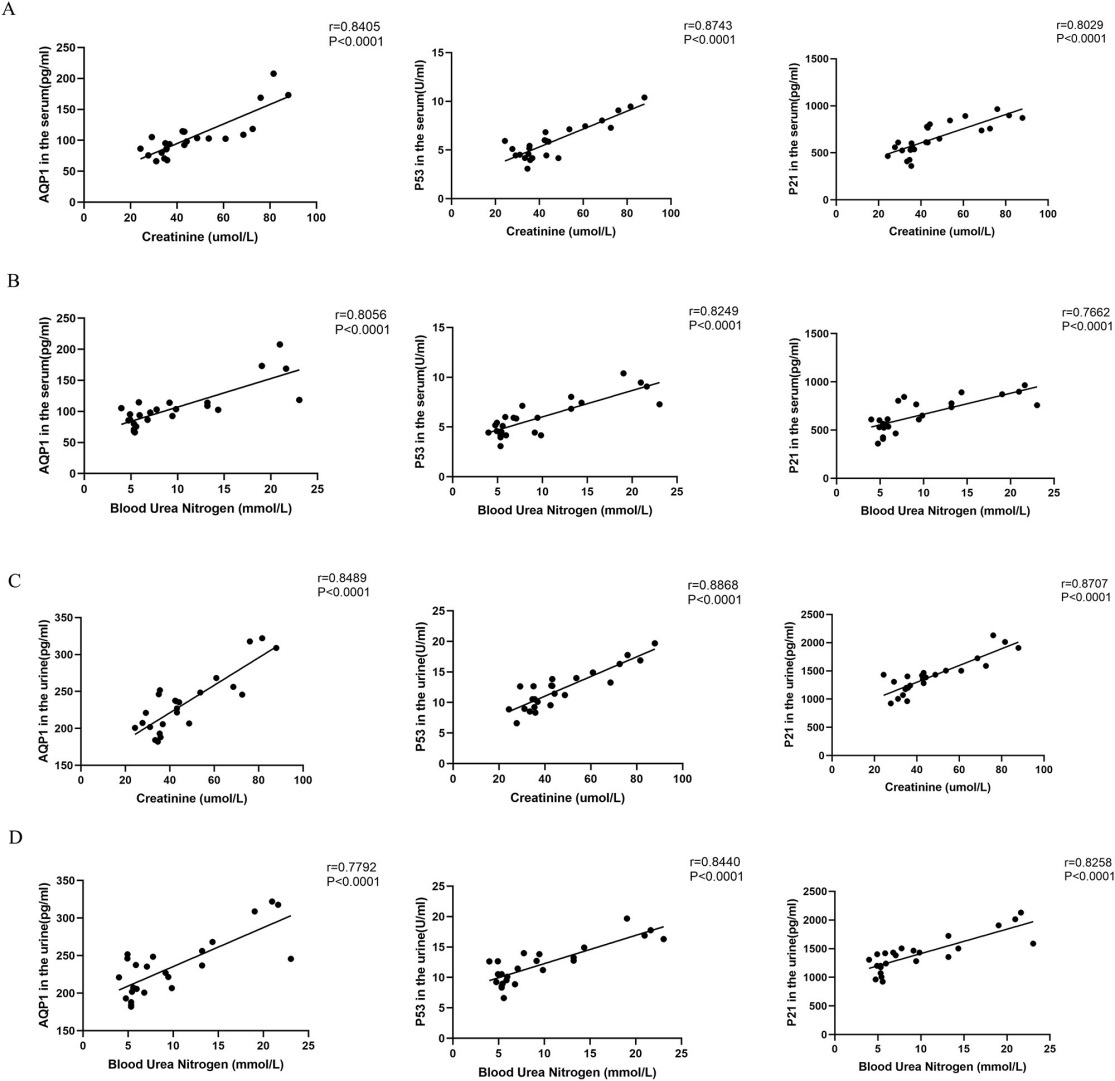
Correlation analysis of SCr and BUN with AQP1, P53 and P21 proteins in serum and urine. (A) Correlation analysis of SCr with AQP1, P53 and P21 in serum. (B) Correlation analysis of BUN with AQP1, P53 and P21 in serum. (C) Correlation analysis of SCr with AQP1, P53 and P21 in urine. (D) Correlation analysis of BUN with AQP1, P53 and P21 in urine.

Pearson correlation analysis was used to explore the correlation between SCr and BUN with AQP1, P53 and P21 proteins in serum and urine at different times in LPS-induced AKI. As shown in (Fig 8A and 8B), AQP1, P53 and P21 protein levels in serum were significantly positively correlated with SCr (r = 0.8405, *P* < 0.0001; r = 0.8743, *P* < 0.0001; r = 0.8029, *P* < 0.0001) and BUN (r = 0.8056, *P* < 0.0001; r = 0.8249, *P* < 0.0001; r = 0.7662, *P* < 0.0001) during the dynamic process of septic AKI. Similarly, the expression of AQP1, P53 and P21 in urine was also significantly positively correlated with SCr (r = 0.8489, *P* < 0.0001; r = 0.8868, *P* < 0.0001; r = 0.8707, *P* < 0.0001) and BUN (r = 0.7792, *P* < 0.0001; r = 0.8440, *P* < 0.0001; r = 0.8258, *P* < 0.0001) (Fig 8C and 8D).

### Correlation analysis of SCr and BUN with AQP1 mRNA, P53 mRNA and P21 mRNA in kidney

**Fig 9:**
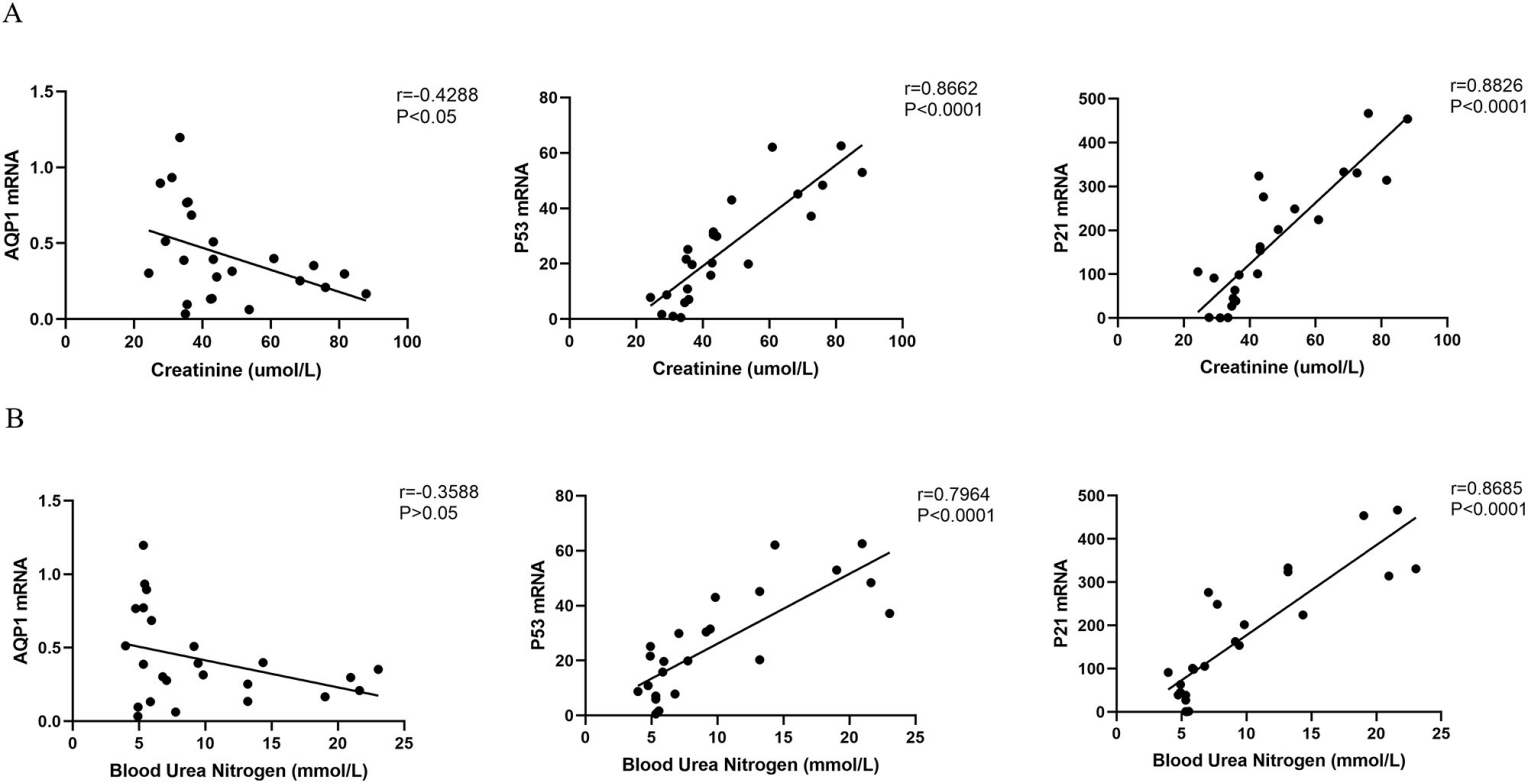
Correlation analysis of SCr and BUN with AQP1 mRNA, P53 mRNA and P21 mRNA in kidney. (A) Correlation analysis of SCr with AQP1 mRNA, P53 mRNA and P21 mRNA in rat kidney. (B) Correlation analysis of BUN with AQP1 mRNA, P53 mRNA and P21mRNA in rat kidney.

As shown in (Fig 9A and 9B), Pearson correlation analysis showed that renal P53mRNA and P21mRNA expression were significantly positively correlated with SCr and BUN levels, respectively (r = 0.8662, *P* < 0.0001; r = 0.8826, *P* < 0.0001; r = 0.7964, *P* < 0.0001; r = 0.8685, *P* < 0.0001). However, AQP1mRNA expression was negatively correlated with SCr and BUN (r = -0.4288, *P* < 0.05) or no correlation (r = -0.3588, *P* > 0.05) (Fig 9A and 9B).

### The ROC curve of serum AQP1, P53, P21 and urinary AQP1, P53, P21 as well as Cr, BUN in LPS induced AKI

**Fig 10:**
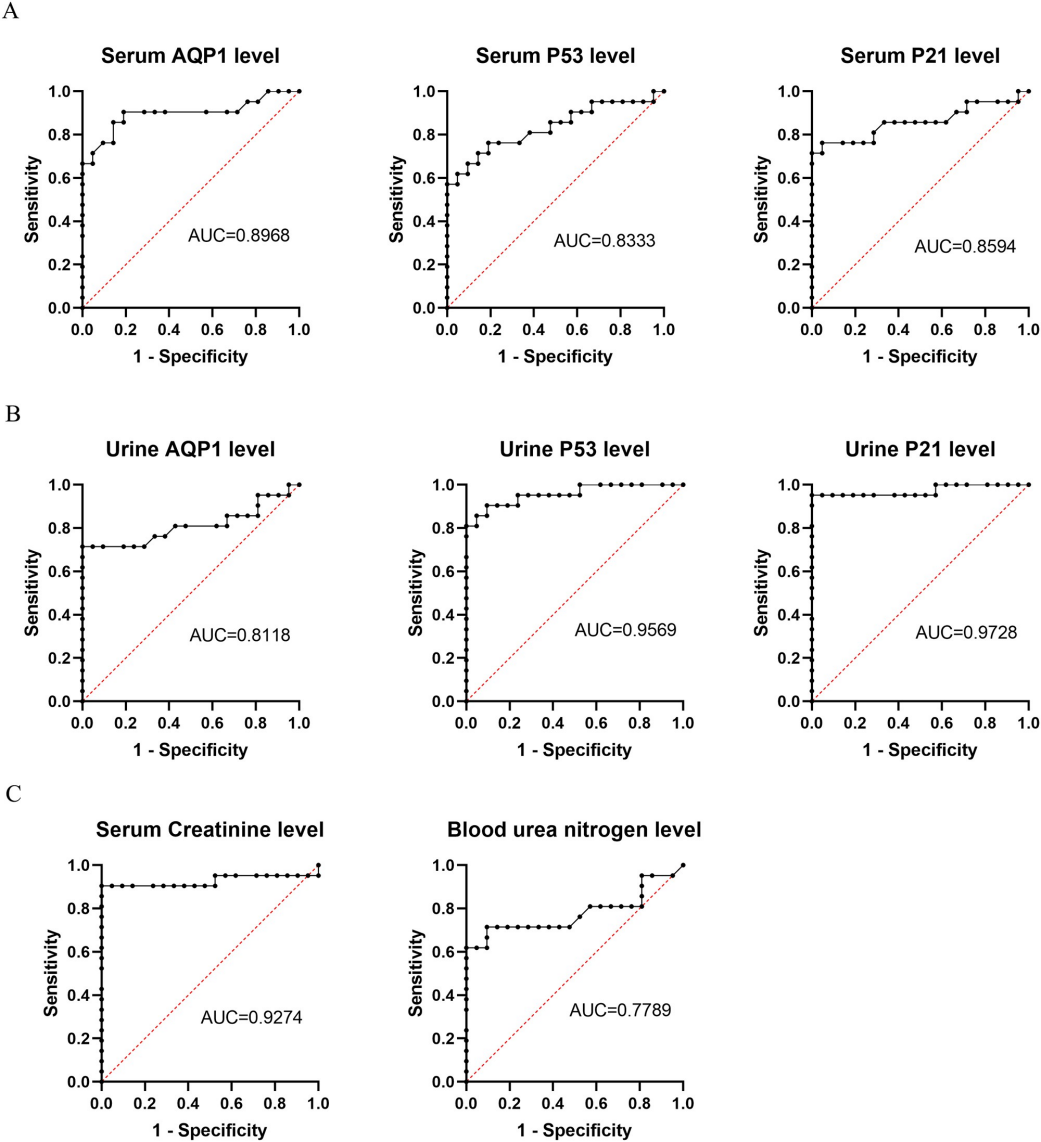
The ROC curve of serum AQP1, P53, P21 and urinary AQP1, P53, P21 as well as Cr, BUN in LPS induced AKI. (A) The ROC curve of serum AQP1, P53 and P21 in LPS induced AKI; (B) The ROC curve of urinary AQP1, P53 and P21 in LPS induced AKI; (C) The ROC curve of Cr and BUN in LPS induced AKI.

To explore more sensitive and specific biomarkers to diagnose AKI, we used a ROC curve assay to investigate the cutoff value of serum AQP1, P53, P21 and urinary AQP1, P53, P21 as well as Cr and BUN levels. The AUCs of serum AQP1, P53, P21 and urinary AQP1, P53, P21 were 0.8968, 0.8333, 0.8594, 0.9569, 0.9728 and 0.8118, respectively (Fig 10A and 10B), which were all better than that of BUN (0.7789) (Fig 10C), and indicated that serum AQP1, P53, P21 and urinary AQP1, P53, P21 could be diagnostic markers of sepsis-AKI with better sensitivity and specificity than BUN.

## Discussion

Currently, the diagnosis of AKI is still based on the 2012 Kidney Disease: Improving Global Outcomes (KDIGO) clinical practice guideline, which states that AKI is diagnosed by a sudden decrease in glomerular filtration rate and SCr increase of ≥50% within 7d; or SCr increase of ≥0.3mg/dL (≥26.5μmol/L) within 48h; or urine volume less than 0.5ml/kg for more than 6h [24]. SCr increased only when the degree of renal injury reaches 50%, but by this time irreversible damage has already occurred in the kidney, indicating that SCr and urine volume are highly compensatory [24, 25]. In other words, SCr and urine volume are highly compensatory. In addition, SCr and urine volume are also susceptible to individual factors. Therefore, there is a certain lag in the diagnosis and treatment of AKI in clinical practice, which brings great suffer and economic burden to patients [26]. At present, numerous researchers are committed to finding new markers for diagnosis of AKI to replace SCr and urine volume, so as to achieve the purpose of diagnosis and prevention of the occurrence and development of AKI.

There are several explanations for the increased expression of AQP1, P53, and P21 in urine: 1) Proximal tubule "stress" proteins, which are upregulated in the early stage of AKI and released into the urinary space [27]. Wang Y [28] et al. showed in vitro that AQP1 genes and proteins expression decreased when LPS stimulated human proximal tubule cell line (HK-2 cells), and overexpression of AQP1 could significantly reduce LPS-induced apoptosis of HK-2 cells. Li B [19] and Liu C [29] et al. demonstrated in vivo experiments that the expression of AQP1 protein in renal tissue was significantly increased after 24h of LPS treatment compared with the control group, which was consistent with our experimental results. These findings suggest that AQP1 is involved in the pathophysiology of septic AKI and plays a nephroprotective role. Moreover, researches have shown that P53 deacetylation alleviates in septic AKI by promoting autophagy [30]. Homsi E [31] et al. and Li C [32] et al. demonstrated in an experimental AKI model that elevated P53 mediated oxidative stress, apoptosis and fibrosis by upregulating P21, thereby aggravating renal tissue injury and the transition to CKD after AKI. Ying Y [10] et al. have confirmed that specific deletion of P53 in mouse proximal tubular cells protects kidney function and histological deterioration after ischemic renal injury (IRI) through reducing necrosis, apoptosis, and inflammation, and also regulates long-term sequelae of IRI via preventing interstitial fibrosis. These results have been demonstrated that inhibition of P53 attenuates proximal tubular epithelial cell apoptosis, inflammatory factor secretion, and oxidative stress in a rat AKI model induced by ischemia-reperfusion. However, some investigators [33] also found that inhibition or knockdown of P53 exacerbated renal tissue injury by promoting inflammatory responses in an ischemia-reperfusion-induced mouse model of AKI. Combining these two types of completely opposite experimental results, we speculate that it may be due to the different species of animals that ultimately lead to completely opposite experimental data and results. Summarizing all findings, it was found that the expression of P53 and P21 in renal tissue significantly increased when AKI was induced by different stimuli. However, contrary to the renal protective effect of AQP1, upregulation of P53 and P21 in the kidney mainly promoted renal tissue injury. Thus, renal P53 and P21 protein were significantly upregulated to promote tissue injury during LPS-induced AKI in rats, while AQP1 protein stress was increased and peaked to play a protective role in the kidney. Meanwhile, a large amount of AQP1, P53 and P21 were released into urine. Finally, the expression of AQP1, P53 and P21 in urine is increased. Besides, the levels of AQP1, P53 and P21 in urine decreased at (24h, 48h, 72h and 7d) after LPS treatment, mainly due to massive consumption of AQP1 in kidney to mitigate the injury and alleviate the post-AKI stress response, which ultimately promote renal tissue repair.2) Low molecular weight plasma proteins that freely filter the glomerular filtration membrane escape the normal tubule reabsorption process due to tubular injury [34]. The contents of serum AQP1, P53, and P21 protein are elevated in septic AKI, and a large amount of AQP1, P53 and P21 proteins filtered from the glomerular filtration membrane and exceeded the reabsorption capacity of renal tubules, resulting in elevated levels of AQP1, P53 and P21 in urine. 3)Proteins from the cytoplasm of tubular epithelium are released into the urine after proximal tubule injury [35]. In the kidney, AQP1 is mainly expressed in the apical and basement membranes of proximal tubular epithelial cells [36], while P53 and P21 are expressed in the cell and nucleus [9]. Thus, a large number of proximal tubular epithelial cells were destroyed in LPS induced-AKI, resulting in the release of AQP1, P53 and P21 proteins in the membrane and cytoplasm of tubular epithelial cells into urine and excreted in vitro. In contrast, the surviving renal tubular epithelial cells migrated and proliferated to repair the damaged renal tubules at (24h,48h,72h,7d) after LPS treatment, which eventually caused the decrease of AQP1, P53 and P21 protein expression in urine. 4)Renal tissue injury stress and severe destruction of proximal tubular epithelial cells as well as renal tubular reabsorption disorder occur simultaneously in septic AKI, which together lead to increased expression of AQP1, P53 and P21 in urine.

However, patients usually show multiple organ damage when sepsis-induced injury occurs. Therefore, we hypothesized that the expression of AQP1, P53 and P21 in serum of rats stimulated by LPS could be increased not only from kidney, but also from other tissues and organs. The results showed that the gene and protein expressions of AQP1, P53 and P21 were significantly increased in heart at(8h,12h,24h,48h) after LPS treatment and decreased at (72h,7d,14d) after LPS treatment. Johnson AC [37] et al. found that during AKI, in addition to the increased expression of P21 in plasma, urine and kidney tissues, which can be used as potentially useful markers of AKI. Concurrently, the expression of P21 in heart and brain was also significantly increased compared with the control group, indicating that AKI can activate P21 in extra-renal organs [37]. Montiel V [38] et al. showed that up-regulation of AQP1 expression in heart aggravates cardiac hypertrophy by mediating hydrogen peroxide delivery, while deletion of AQP1 or selective blocking of AQP1 subunit pores can inhibit H_2_O_2_ transport in mouse and human cells and rescue cardiac hypertrophy in engineered mouse and human IPSC-derived cardiomyocytes. These results suggest that AQP1 is up-regulation in cardiac hypertrophy or cardiomyocyte injury, which consistent with our experimental results. Furthermore, Su X [39] et al. and Han G [40] et al. revealed that AQP1 expression was decreased in lung tissues with LPS-induced acute lung injury. W. Liang [41] and colleagues have shown that AQP1 expression is down-regulated in lung tissue during CLP-induced acute lung injury in rats. Besides, there is evidence that AQP1 localizes to lymphatic endothelial cells throughout the gastrointestinal submucosa [42] and lamina propriaand AQP1-null mice have defects in fat absorption [43]. Therefore, AQP1 might be involved in the fat digestion process. These findings support our experimental results that the mRNA and protein levels of AQP1, P53 and P21 in lung and small intestine were significantly decreased at (8h,12h,24h,48h) after LPS treatment and recovered at 72h,7d and14d. Therefore, we found that the increased expression of serum AQP1, P53 and P21 protein in LPS treatment for (8h,12h,24h,48h) may originate from both kidney and heart but not lung and small intestine by summarizing the results of proven studies and combining our experimental findings.

NGAL, also known as lipocalin-2, was first discovered in the study of Neutrophil gelatinases and is a member of the lipocalin family, which molecular weight is about 25KD [44, 45]. Subsequently, the scientists found that NGAL not only exists in neutrophils, also can appear in the epithelial cells of a particular [46], when ischemic or toxicity of kidney injury, renal tubular epithelial cells of NGAL expression increased, urine and NGAL levels were elevated in the blood. Therefore, NGAL is early markers of stress and damage of acute kidney injury [47]. KIM-1 is a type 1 transmembrane glycoprotein that is present in low concentrations in normal renal tissue or urine but expressed at high levels in proximal tubular epithelial cells dedifferentiated after renal ischemia [48]. A soluble form of human KIM-1 can be detected in the urine of patients with acute tubular necrosis and may therefore serve as a useful biomarker of proximal tubular injury [49]. At present, a number of studies have proved that the expression of NGAL and KIM-1 is increased in various types of AKI, which is often used as an early diagnostic molecule for renal injury and stress [50–53]. As expected, renal NGAL and KIM-1 gene levels increased sharply at 8h and12h after LPS treatment and gradually decreased at (24h,48h,72h,7d,14d), indicating that 12h of LPS treatment was the most severe period of renal tissue injury, and (24h,48h,72h,7d,14d) of LPS treatment were the stages of renal repair and recovery after AKI. Surprisingly, NGAL mRNA expressions in heart, lung and small intestine were significantly enhanced in LPS treatment for (8h,12h,24h,48h) and recovered at 72h,7d and14d. However, in addition to the kidney, the expression of KIM-1 mRNA in other tissues and organs, mainly including the heart, lungs and small intestine, did not change during the process of LPS stimulation in rats. There are the following explanations: 1) LPS treatment of rats (8h,12h,24h) induced systemic inflammatory response contributes to the rapid and large accumulation of neutrophils in kidney, heart, lung and small intestine, thereby increasing the expression of NGAL. Subsequently, the body reduced the inflammatory response through autoimmune regulation mechanism at (48h,72h,7d,14d) after LPS treatment. The neutrophils’ mission has been accomplished, resulting in decreased expression of both neutrophils and NGAL in the tissue. 2) The expression of NGAL in organs is changed. Since Bundgaard JR [44] et al. discovered NGAL in human neutrophils, J. B. Cowland et al. [46] further demonstrated the differential expression of NGAL gene in various human tissues and organs. This is consistent with our results demonstrating NGAL gene expression in kidney, heart, lungs, and small intestine of rats not treated with LPS. Furthermore, research have found that NGAL plays an anti-inflammatory and anti-injury protective role [54]. Therefore, the expression of NGAL in organs (kidney, heart, lung, and small intestine) significantly increased to play an anti-inflammatory and anti-injury role at (8h,12h,24h) after LPS treatment, while the organ damage was alleviated during the recovery period at (48h,72h,7d,14d), contributing to the expression of NGAL was significantly decreased. These results indicated that NGAL could reflect early renal injury without specificity, while KIM-1 could indicate both specificity and early renal injury. In other words, NGAL is similar to an acute stress molecule, and its expression will increase as long as the tissue is damaged by stimulatory factors, suggesting that NGAL may serve as an indicator of sepsis-induced multiple organ injury.

## Conclusion

In conclusion, our results showed that the expression of AQP1, P53 and P21 in serum, urine and kidney were significantly elevated first and then gradually decreased in LPS induced-AKI of rats, which suggested that AQP1, P53 and P21are involved in the pathological process of AKI and may be used as early diagnostic markers of AKI in sepsis. Moreover, AQP1, P53, P21 and NGAL were differentially expressed in different tissues and organs (in heart, lung and small intestine), but the expression of KIM-1 mRNA was not affected. NGAL may be used as indicators of sepsis-induced multiple organ damage during the process of LPS-induced AKI. However, the specific mechanism of AQP1, P53, P21 and NGAL changes in LPS-induced AKI or systemic organ damage still needs further exploration and research in the future.

## Supporting information

S1 File (ZIP)
